# The Impact of COVID-19 Pandemic on Respiratory Syncytial Virus Infection in Children

**DOI:** 10.1155/2024/2131098

**Published:** 2024-10-18

**Authors:** Jose Boris Trigueros Montes, Diego Montes, Andrew Miele, Won Baik-Han, Gagan Gulati, Lily Q. Lew

**Affiliations:** ^1^Department of Pediatrics, Flushing Hospital Medical Center, Flushing, New York 11355, USA; ^2^Department of Biology, Baylor University, Waco, Texas 76706, USA; ^3^Department of Research Education and Innovation, Medisys Health Network, Jamaica, New York 11418, USA

**Keywords:** COVID-19 pandemic, infection, pediatric, respiratory syncytial virus

## Abstract

**Background/Objective:** Respiratory syncytial virus (RSV) is a major cause of bronchiolitis in infants and young children. Bronchiolitis, an acute inflammation of the lower respiratory tract, can lead to pneumonia, respiratory failure, and death. We aimed to compare the incidence and severity of RSV infection in children aged 0–60 months before and during the coronavirus disease 2019 (COVID-19) pandemic.

**Methods:** A retrospective chart review was conducted on patients aged 0–60 months who tested positive for RSV between May 1, 2018, and May 31, 2022, in a community hospital in Queens County, New York City, United States. Comparisons were made between seasons 2018–2019 and 2019–2020 as before, and seasons 2020–2021 and 2021–2022 as during the COVID-19 pandemic. Severity of RSV infection was assessed using the Bronchiolitis Severity Score (BSS). Data were analyzed using R software, a *p* value of < 0.05 was considered statistically significant.

**Results:** The incidence of RSV infection in seasons 2018–2019 and 2019–2020 peaked from mid-October to February, whereas the first season during the COVID-19 pandemic (2020–2021) was truncated with a very low incidence of RSV infection, and season 2021–2022 peaked from September to January, with the highest incidence (37%) and lower frequency of RSV infection at any given point. Patients during the season 2021–2022 were older (*H* [2, 196.6] = 12.5, *p* < 0.001, 95% *CI* = [5.4, 25.6]) and had milder illness (*H* [2, 187.5] = 7.5, *p* < 0.01, 95% *CI* = [2.1, 19.2]).

**Conclusions:** We observed a lower incidence of RSV infection and a lower rate of hospitalization for RSV during the COVID-19 pandemic. The second RSV season during the COVID-19 pandemic began earlier, lasted longer, and had a lower frequency. Older children with milder illnesses were affected most during this season. RSV epidemiology and disease burden were impacted by the COVID-19 pandemic and could have significant ramifications for its prevention and control strategies.

## 1. Introduction

Respiratory syncytial virus (RSV) infection occurs frequently and is a major cause of bronchiolitis and pneumonia in infants and children [1]. In the United States, RSV infection accounts for more than 57,000 hospitalizations and 500,000 emergency department visits annually in children younger than 60 months [2]. In regions with a temperate climate such as that in New York City, United States, the RSV season traditionally occurs from mid-October to early May with a median national peak in early February [3]. Climate changes with warmer and wetter weather can alter the seasonality and RSV infection. Most infants and children develop a mild, self-limited illness lasting 1–2 weeks; however, more severe presentations can occur [4]. The disease burden of RSV infection is a consequence of limited preventive strategies. Risk factors for severe illness include prematurity, chronic lung disease, congenital heart disease, weakened immune system, and neuromuscular disorders [5]. A monoclonal antibody was approved for these high-risk infants and children only, especially when a vaccine was not available and when significant adverse effects accompanied the use of the only antiviral agent for RSV. When bronchiolitis occurs, severity of the illness can be assessed using the Bronchiolitis Severity Score (BSS). Of the many clinical scoring tools for bronchiolitis, BSS has a better interobserver agreement of physical signs [6]. After the World Health Organization (WHO) declared the novel coronavirus disease of 2019 (COVID-19) a global pandemic in March 2020, New York City closed schools and nonessential businesses, mandated mask wearing, and imposed social distancing [7]. These measures were expected to curb the exposure to and the transmission of the severe acute respiratory syndrome coronavirus 2 (SARS-CoV-2) and to ultimately result in the reduction in viral illnesses. Within days, New York City became the initial epicenter in the United States with significant reduction in pediatric emergency department visits for fear of exposure to the SARS-CoV-2 virus and curtailed accessibility to medical care due to overwhelmed health care systems. With the passage of the COVID-19 pandemic, we report the impact of COVID-19 on the incidence and severity of RSV infection before and during the pandemic. Health care providers and political decision makers can utilize the observed changes in RSV epidemiology as seen during the pandemic to optimize future prevention strategies, to improve health care system preparedness, and to reduce disease burden. This community hospital–based study highlighting the seasonal changes and atypical spike can provide the added evidence needed to reconcile conflicting observations in the care of young children who are the most vulnerable to severe RSV infections.

## 2. Material and Methods

**Figure 1:**
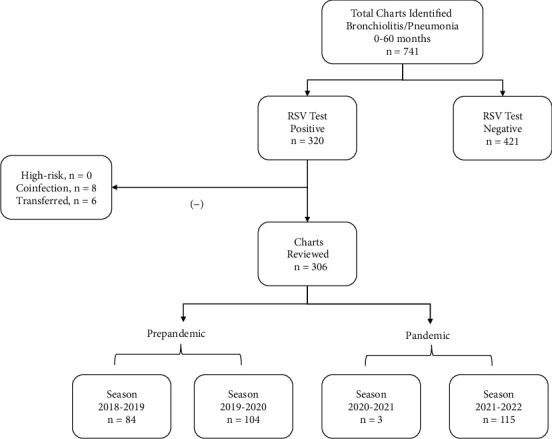
Flow diagram of patients reviewed before and during the COVID-19 pandemic.

We reviewed charts of patients aged 0–60 months seen in an urban 293-bed not-for-profit teaching hospital pediatric emergency department between May 1, 2018, and May 31, 2022, who tested positive on either a rapid RSV antigen test or a polymerase chain reaction for RSV. The Institutional Review Board approved the study. The *International Classification of Diseases, Tenth Revision*, codes B97.4, J21.0, and J12.1 were used to identify patient charts. Children with risk factor(s) for severe illness or coinfection or who were transferred to an intensive care unit were excluded from the study (see Figure 1). Data extracted from electronic health records included date of visit, demographics, clinical presentation, disposition, and length of stay (LOS). Demographics included age, gender, and ethnicity. BSS–evaluating respiratory rate, retractions, wheezing on auscultation, and general condition were determined for each case. Of a possible score of 0–12, a score of 0–3 represented mild illness, 4–6 moderate illness, and greater than 6 severe illness (see Supporting Information (available here)). One author performed the BSS calculations. Missing data were not input into the analyses. Seasons 2018–2019 and 2019–2020 were considered as before or reference years, and Seasons 2020–2021 and 2021–2022 as the first and second years during the COVID-19 pandemic, respectively. Incidence, defined as the number of positive cases, and severity, defined by the BSS, were compared between the RSV seasons. Data were analyzed using R software, Version 4.2.2. Demographic characteristics were compared between the seasons. Chi-square tests and Cramer's V were used to test differences in categorical variables including the proportion of cases by seasons. For comparisons of nominal variables, Cramer's V was used to estimate the effect size. Nonparametric equivalents were used when appropriate based on variable type and distribution. A *p* value of < 0.05 was considered statistically significant. The STROBE reporting guidelines were followed.

## 3. Results

**Figure 2:**
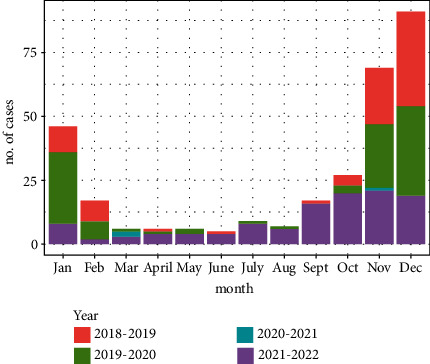
The number of respiratory syncytial virus rapid antigen test or polymerase chain reaction positive patients per month per year seen in the pediatric emergency department between 2018 and 2022. The highest incidence per month was seen in Season 2021–2022.

A total of 741 charts were identified. Of the 306 (41%) patients who met the inclusion criteria of age between 0 and 60 months and having a RSV positive test, half were male (51%), and the most prevalent ethnicity was Hispanic (74%) followed by Asian (24%), reflective of the community. Season 2018-2019 (27%) and Season 2019–2020 (35%) followed the traditional mid-October to early May pattern. Season 2020–2021 had the lowest incidence (1%), and Season 2021–2022 had the highest incidence (37%), (see Table 1). Differences in incidence across seasons were significant (*χ*^2^ [4] = 606, *p* < 0.001). Cases in Season 2021–2022 presented in September lasted over a longer period of time and had a lower frequency at any given point (see Figure 2). In the same season, the patients were older (*H* [2, 196.6] = 12.5, *p* < 0.001, 95% CI = [5.4, 25.6]) and had milder illness (*H* [2, 187.5] = 7.5, *p* < 0.01, 95% CI = [2.1, 19.2]) compared to patients seen in previous seasons. Number of hospitalizations (Season 2020–2021 was excluded from significant analysis due to low incidence) declined the least in Season 2021–2022 (*χ*^2^ [2] = 17.2, *p* < 0.001, *V* = 0.18, 95% CI = [0.1, 0.3]), and the median LOS was 3 days, (IQR = 2, *H* [2, 89.9] = 0.005, *p* = 0.95, 95% CI = [0.03, 4.4]).

## 4. Discussion

RSV, a highly contagious respiratory virus among young children, spreads through droplets from the nose and throat of infected individual's cough and sneeze, either from direct contact or on contaminated surfaces [1]. Although most infants with RSV infection develop a mild, self-limited illness lasting 1–2 weeks, bronchiolitis and pneumonia can occur in children younger than 1 year of age leading to respiratory failure, apnea, and death [2]. The disease burden in the United States for children aged 0–60 months includes over two million outpatient visits, 58,000–80,000 hospitalizations, and 100–300 deaths annually [3]. The typical symptoms of RSV infection include rhinorrhea, cough, fever, and decreased appetite [4]. Similar to influenza and SARS-CoV-2, RSV typically circulates in the late fall and extends into the spring months in temperate climate regions making diagnosis difficult [8, 9]. To detect RSV infection, rapid antigen testing and polymerase chain reaction specific for RSV or in combination with influenza and SARS-CoV-2 are available. Viral seasons and circulation patterns are monitored globally by the WHO, nationally by the National Respiratory and Enteric Virus Surveillance Systems (NREVESS), and additionally at regional and state levels in the United States [10]. Awareness of the incidence and seasonality of RSV helps guide the timing of immunoprophylaxis and health care utilization during anticipated peaks. Our study, set in a multiethnic community hospital in New York City, demonstrated the atypical nature of RSV seasons during the COVID-19 pandemic and the differences in severity as determined by the BSS when preventive strategies were limited. The data assembled in this report can add to the growing body of knowledge on the changing epidemiology to better understand the future of RSV infection.

We observed a lower number of RSV cases and lower rate of hospitalization for RSV during the first season of the COVID-19 pandemic followed by a higher number of RSV cases in the second season. Many other groups reported this unusual pattern as well [11–14]. This significant difference in the proportion of cases was attributed to the imposed interventions to slow the spread of the SARS-CoV-2 virus and to the changes in health care-seeking behaviors in the first season during the COVID-19 pandemic. With the relaxation of mask wearing and restricted activities, the incidence of RSV returned to previous levels and shifted to an atypical pattern [15]. In addition, the approval of the COVID-19 vaccine for use in children aged 6–60 months was also delayed resulting in their incomplete return to school and day care. The increase in incidence seen in the second season could be underestimated by this staggered return to normalcy.

Globally, studies from the United States, Australia, Japan, and England all demonstrated a decline in RSV illness during the first season followed by a robust rebound in incidence during the second season of the pandemic [16–21]. Both a multicenter database review by Wang et al. and a single-center study by Rao et al. in the United States with larger sample sizes concurred with our unusual surge of RSV infection in children aged 0–60 months by the second RSV season during the COVID-19 pandemic [17, 18]. Besides observing a change in RSV epidemiology, Foley et al. perceived almost three times the number of RSV infections during Australia's equivalent COVID-19 pandemic second season compared to prepandemic RSV peak months [19]. Ujiie et al. compared five seasons, beginning a year earlier than our study, and reported a 17-fold cumulative increase in their second season, higher and earlier than the typical summer and autumn seasons in Japan [20]. In England, Bardsley et al. reported a greater reduction in the number of RSV cases during their first winter season compared to the observed return in numbers in the second season accounting for the steep upswing of cases [21]. Furthermore, New York City is experiencing climate changes including warmer temperatures and increased rainfall similar to most of the world [22]. Reports correlated these changes with earlier, less severe, and possibly a bimodal pattern of RSV infection [23, 24]. The ongoing climate changes may already play a role in RSV epidemiology, and its effect will require continued and further surveillance. The nonpharmacological measures did slow the transmission of the SARS-CoV-2 virus as well as reshaped the epidemiology of RSV observed locally and globally.

Children infected with RSV in the second pandemic season studied were older and had milder illness. Despite milder illness, a change in the median LOS was not observed. We limited the children studied to those aged 0–60 months compared to the many other studies that included patients up to age 18 years. Differences in the age distribution of those with RSV infection during the pandemic were observed in the majority of the studies worldwide and were in agreement with our data [13]. Both Rao et al. in the United States and Viñeta Paramo et al. in Canada agreed on higher proportion of older hospitalized children with RSV infection but disagreed on the change in severity during Season 2021–2022 [13, 18]. Foley et al. also observed twice the median age in those infected with RSV during the pandemic compared to the median age before the pandemic in months without differences in severity [19]. Waning immunity and the lack of previous exposure to RSV may contribute to the increase in incidence of RSV infection in older children during the COVID-19 pandemic. Data exist as well implicating an increased risk of RSV infection after COVID-19 infection or immune dysregulation to account for some of the surge after the decline especially in the immediate post-COVID-19 seasons [17, 18]. Both immunity naivety and immunity dysregulation are possible hypotheses for the higher incidence of RSV infection in older children [18, 19]. We did not collect data on SARS-CoV-2 infection in temporal relationship to RSV infection or COVID-19 vaccine rates in those eligible between 6 and 60 months of age. Greater differences and trends in the incidence of RSV infection could be seen had our study continued for an additional season with cessation of masking and social distancing.

Severity of RSV infection depends on the host, environment, and viral factors. Although anticipated, there were no children at high risk for severe disease to exclude. It was also important to know that our high-risk infants were protected through the administration of monoclonal antibodies. Of the demographic and environmental risk factors, male gender, younger than 6 months of age, low socioeconomic status, household crowding, school-age siblings in the household or in day care, duration of breastfeeding less than 2 months, and indoor tobacco smoke exposure have been reported to be associated with serious disease [25]. By age 2 years, almost all children have had at least one RSV infection, and infection reoccurs throughout life with lesser severity until older age with underlying comorbidities [25, 26]. Severity of bronchiolitis can be assessed by tachypnea, retractions, prolonged expiration, wheezing on auscultation, and general condition as described by the BSS [6]. We applied the BSS score to compare the severity of bronchiolitis across seasons (see Table 1). A higher BSS corresponded with increased severity of illness. There are no studies in the literature using both the BSS and the number of hospitalizations to designate disease severity. The number of cases with BSS greater than six was not greater during the COVID-19 pandemic compared to the number of cases before the COVID-19 pandemic. Cases transferred to a pediatric intensive care unit as a measure of increased severity were less than 1% and were not included in the study. Since we are a community hospital without a pediatric intensive care unit, the number of hospitalizations and the number of severe cases may be underestimated. The milder illness in older children can alternatively be explained by their larger airways rather than related to one of two known subtypes, A and B, which we did not stratify. RSV-A has a higher prevalence rate and data support increased or equivalent severity compared to RSV-B [27]. Other than age and gender, we did not assess the impact of other environmental risk factors on severity. The vulnerability of severe illness was in the very young and contributed to most of the disease burden.

Pharmacological preventive strategies against and treatment options for RSV infection were not many at the time of our study. The use of monoclonal antibodies and vaccines is driven by the onset, peak, and duration of seasonality of RSV. Palivizumab, a monoclonal antibody recommended for high-risk infants and young children based on gestational age and certain underlying medical conditions to prevent RSV, was available [28]. Within months after declaring the end of the COVID-19 pandemic in the United States, the United States Food and Drug Administration approved a long-acting monoclonal antibody known as nirsevimab as a single dose by injection to be given to infants younger than 8 months of age born during or entering their first RSV season, and a maternal vaccine for use during 32–36 weeks of pregnancy between September through January as one dose by injection 2 weeks before birth to provide protective proteins to the neonate in the first 6 months after birth [29, 30]. A vaccine in the 1960s and an antiviral agent known as ribavirin in the 1980s both failed to reduce the significant morbidity and mortality caused by RSV [31]. No patient in our study was treated with an antiviral agent. Our study was terminated after two seasons into the pandemic and before the availability of the new pharmacological agents. In a recent systematic analysis, Cong et al. concluded that community–based RSV surveillance is needed when the changes in RSV epidemiology varied globally as countries recover differently from the impact of COVID-19 [32]. We speculate that the newer preventive strategies and the global recovery from the COVID-19 pandemic are expected to change the incidence and severity of this viral illness in the coming seasons. Both nonpharmacological and pharmacological measures are important and necessary to control the spread of respiratory viral illnesses as seen in this community hospital–based study.

The limitations of our study were its retrospective nature and small sample size in a single center. We studied only two seasons before and two seasons during the COVID-19 pandemic. Charts were completed by observers in different levels of pediatric training and attested by an attending physician. As an observational study, there are inherent confounding biases with no intention to demonstrate causality. The strengths of our study include applying a clinical score to evaluate severity besides the number of hospitalizations and categorizing our patients by ethnicity not reported previously. Our additional data can be used to reconcile conflicting observations.

We conclude that the decline in RSV cases in the first season year 2020–2021 during the COVID-19 pandemic was attributed to the imposed interventions to halt the spread of respiratory viruses by temporal concurrence. The higher incidence and earlier peak in the second season of the COVID-19 pandemic coincided with the reopening of schools and businesses, the relaxation of mask wearing and social distancing, and before widespread implementation of pharmacological preventive strategies. Patients seen in the 2021–2022 season were older, and the majority had mild illness. Despite milder illness, the median LOS was unchanged. Waning immunity, immune dysregulation, and changes in viral behavior may account for the differences in severity in those infected with RSV during the COVID-19 pandemic. Future monitoring studies will include the effect of newer prevention strategies and environmental factors on the incidence and severity in the coming seasons. Without a RSV vaccine for children aged 0–60 months, RSV disease burden will continue to be high in this age group. Health care providers and political decision makers can access and share data such as those collected in our study to stay abreast with the changing epidemiology and the newer preventive options and implementation strategies with the goal of lowering the morbidity and mortality of this respiratory viral illness.

## Figures and Tables

**Table 1:** Comparing RSV infection before and during the COVID-19 pandemic.

**Variables**	**2018–2019**	**2019–2020**	**2020–2021** ^+^	**2021-2022**	**p** ** value**
Number (%)	84 (27)	104 (35)	3 (1)	115 (37)	<0.001^∗^^a^
Peak months	Oct 2018–Feb 2019	Oct 2019–Feb 2020	Mar 2020–May 2021	Sept 2021–Jan 2022	
Age (months) mean (SD)	8.6 (7.3)	9.3 (8.1)	3.3 (2.1)	14.7 (10.7)	<0.001^b^
BSS (n) %					<0.01^a^
0–3	54 (64)	64 (62)	2 (67)	90 (78)	
4–6	24 (29)	24 (23)	1 (33)	20 (17)	
> 6	6 (7)	16 (15)	0	5 (4)	
BSS (median) IQR	2 (0, 5)	2 (0, 5)	3 (2, 5)	0 (0, 3)	< 0.01^b^
Hospitalization (*n*) %	53 (63)	53 (51)	0	39 (34)	< 0.001^∗^^a^
LOS median (IQR)	3 (2, 4)	3 (2, 4)	4 (3, 5)	3 (2, 4)	0.95^b^

Abbreviations: BSS: Bronchiolitis Severity Score, IQR: interquartile range, LOS: length of stay, SD: standard deviation.

^+^Season 2020–2021 was not included in the analysis.

^a^Chi-square test.

^b^Kruskal–Wallis test.

^*^ *p* <0.05 was considered statistically significant.

## Data Availability

The data used to support the findings of this study were included within the article.

## Supporting Information

Supporting Information Additional supporting information can be found online in the Supporting Information section. Bronchiolitis Severity Score (adapted from Chin et al.)^6^ was used to evaluate four physical variables for each case.
