# Supplementary material for: The Impact of COVID-19 Pandemic on Respiratory Syncytial Virus Infection in Children
Source: Pulm Med. 2024 Oct 18;2024:2131098. doi: 10.1155/2024/2131098 (PMC11511594; doi:10.1155/2024/2131098)
Supplement: Supporting Information — Additional supporting information can be found online in the Supporting Information section. Bronchiolitis Severity Score (adapted from Chin et al.)6 was used to evaluate four physical variables for each case. [file 2131098.f1.pdf]

Supplementary material

Bronchiolitis Severity Score (adapted from Chin et al.)[6]

Variables	0 point	1 point	2 points	3 points
Respiratory rate (breaths/minute)	<30	30-45	46-60	>60
Retractions	None	Intercostal	Intercostal<br>Substernal	Intercostal<br>Substernal<br>Supraclavicular
Auscultation (wheezing)	None	End expiratory	Expiratory	Inspiratory<br>Expiratory
General condition	Normal			Irritable<br>Lethargic<br>Poor feeding

Score: 0-3 represent mild illness, 4-6 moderate illness, and greater than 6 severe illness
